# Immunohistochemical and genomic profiles of diffuse large B-cell lymphomas: Implications for targeted EZH2 inhibitor therapy?

**DOI:** 10.18632/oncotarget.3154

**Published:** 2015-02-05

**Authors:** Sydney Dubois, Sylvain Mareschal, Jean-Michel Picquenot, Pierre-Julien Viailly, Elodie Bohers, Marie Cornic, Philippe Bertrand, Elena Liana Veresezan, Philippe Ruminy, Catherine Maingonnat, Vinciane Marchand, Hélène Lanic, Dominique Penther, Christian Bastard, Hervé Tilly, Fabrice Jardin

**Affiliations:** ^1^ INSERM U918, Centre Henri Becquerel, Université de Rouen, IRIB, Rouen, France; ^2^ Department of Pathology, Centre Henri Becquerel, Rouen, France; ^3^ Department of Clinical Hematology, Centre Henri Becquerel, Rouen, France

**Keywords:** DLBCL, EZH2, Methylation, Immunohistochemistry, NGS

## Abstract

Enhancer of Zeste Homolog 2 (EZH2) plays an essential epigenetic role in Diffuse Large B Cell Lymphoma (DLBCL) development. Recurrent somatic heterozygous gain-of-function mutations of EZH2 have been identified in DLBCL, most notably affecting tyrosine 641 (Y641), inducing hyper-trimethylation of H3K27 (H3K27me3). Novel EZH2 inhibitors are being tested in phase 1 and 2 clinical trials but no study has examined which patients would most benefit from this treatment. We evaluated the immunohistochemical (IHC) methylation profiles of 82 patients with DLBCL, as well as the mutational profiles of 32 patients with DLBCL using NGS analysis of a panel of 34 genes involved in lymphomagenesis. A novel IHC score based on H3K27me2 and H3K27me3 expression was developed, capable of distinguishing patients with wild-type (WT) EZH2 and patients with EZH2 Y641 mutations (*p* = 10^−5^). NGS analysis revealed a subclonal *EZH2* mutation pattern in EZH2 mutant patients with WT-like IHC methylation profiles, while associated mutations capable of upregulating EZH2 were detected in WT EZH2 patients with mutant-like IHC methylation profiles. IHC and mutational profiles highlight *in vivo* hyper-H3K27me3 and hypo-H3K27me2 status, pinpoint associated activating mutations and determine *EZH2* mutation clonality, maximizing EZH2 inhibitor potential by identifying patients most likely to benefit from treatment.

Diffuse large B-cell lymphoma (DLBCL) is the most common lymphoid malignancy, accounting for 30–40% of all Non Hodgkin Lymphomas (NHL) [1]. Gene expression profiling has identified two main subtypes: Germinal Center B-cell like (GCB) and Activated B-Cell like (ABC), with the ABC subtype having the most unfavorable prognosis [2, 3]. The development of immuno-chemotherapy, and most notably rituximab, has revolutionized the standard-of-care treatment of DLBCL but a large part of patients still relapses or is refractory to treatment.

Recently, epigenetic regulation has been shown to be a crucial element in DLBCL development, and gene repression mediated by Polycomb Repressive Complexes 1 and 2 (PRC1 and PRC2) has garnered attention. Enhancer of Zeste Homolog 2 (EZH2), the catalytic subunit of PRC2 [4], is a histone methyl-transferase capable of specifically mono-, di- and tri-methylating histone H3 lysine 27 (H3K27me1, H3K27me2, and H3K27me3) [5].

Recurrent somatic heterozygous gain-of-function mutations of EZH2 have been identified in DLBCL, most notably affecting tyrosine 641 (Y641), inducing increased H3K27me3 [6, 7]. More recently, multiple studies have shown cell lines with *EZH2* mutations to be dependent on the higher catalytic activity of mutant EZH2 Y641 for proliferation, leading to the development of novel EZH2 inhibitors for therapeutic use, capable of reversing malignant phenotype [8–11].

Two EZH2 inhibitors are currently being tested in phase 1 and 2 clinical trials both in patients with and without EZH2 Y641 mutations (NCT01897571 and NCT02082977), but no study has specifically examined which patients would be most susceptible to benefit from this treatment and how to screen for them. Patients with EZH2 gain-of-function mutations have been pinpointed as ideal EZH2 inhibitor recipients [8–11]; nevertheless, in today's targeted therapy era, it seems essential to establish a method of detecting optimal candidates for EZH2 inhibitor treatment.

In the current study, we examined whether a simple immunohistochemical (IHC) technique could be used to distinguish wild-type (WT)-like and mutant-like EZH2 IHC methylation profiles, and thus screen for patients with confirmed overactive EZH2 at the protein level. We also used Next Generation Sequencing (NGS) analysis to further detail patients' genomic profiles and to determine whether associated mutations could justify EZH2 inhibitor treatment for patients otherwise not considered. We propose that these methods, used in conjunction, could serve to better determine candidates most likely to respond to EZH2 inhibitor treatment.

## MATERIALS & METHODS

### Patients and biological samples

96 patients with *de novo* DLBCL at diagnosis with available tumor DNA and Formalin-Fixed Paraffin-Embedded (FFPE) samples were included for *EZH2* Sanger sequencing analysis and subsequent immunohistochemistry experiments. To provide a comprehensive genomic description of DLBCL, targeted NGS experiments were performed in 32 patients (20/96 and 12 additional cases not in our initial cohort). A flowchart summarizes the experimental methods used on the entire cohort (Supplementary Figure 1). Table 1 summarizes the patients' clinical characteristics. Median follow-ups for overall survival and progression-free survival were respectively 4.9 and 3.9 years. All experiments were in accordance with the Helsinki Declaration and the study was approved by the internal review board.

**Table 1:** Clinical characteristics of patients at diagnosis

Clinical parameter	Patients at diagnosis (*n* = 96)
Gender M/F, *n*	48/48
Age (years), *median (range)*	66 (17–87)
Adverse prognostic factors, *n* (%)	
Age > 60 years	60 (63)
Ann Arbor stage III–IV	68 (71)
LDH > N	9 (9)
Extranodal sites ≥ 2	37 (39)
Bulky mass ≥ 10 cm	20 (21)
Performance status ≥ 2	26 (27)
IPI, *n* (%)	
0–2	42 (44)
3–5	54 (56)
Treatment, *n* (%)	
R-CHOP	38 (40)
R-ACVBP	17 (18)
R-mCHOP	13 (14)
R-IVA	1 (1)

**Abbreviations:** LDH, Lactate Dehydrogenase; IPI, International Prognostic Index; R, Rituximab; CHOP, Cyclophosphamide, Hydroxydaunorubicin, Vincristine and Prednisone; ACVBP, Doxorubicin, Cyclophosphamide, Vindesine, Bleomycin and Prednisone; mCHOP, miniCHOP; IVA, Ifosfamide, Etoposide and Cytarabine

### Immunohistochemistry

Sections from FFPE tissue samples were used to build Tissue Microarrays (TMAs). Information on the primary antibodies used in this study (EZH2, H3K27me1, H3K27me2 and H3K27me3) is summarized in Supplementary Table 1. Deparaffinization, rehydration, and epitope retrieval was performed by PT Link following the manufacturer's instructions at pH 6 (DAKO, California, USA) and deparaffinized sections were stained using Vectastain kits (Vector Laboratories Inc, California, USA) according to the manufacturer's instructions. The slides were then incubated with DAB+ chromogen for 5 minutes and counterstained with hematoxyline for 2 minutes. Slides were scored in a blinded fashion by an experienced anatomopathologist (JMP). Slides were also scored in a blinded fashion by a second independent anatomopathologist (ELV) in order to assess correlation. Cases with lost TMA cores or non-tumoral tissue were excluded. Tumors were scored according to staining intensity (1–3, with 1 being weak and 3 strong) and proportion of tumor cells stained (0–10, with 0 representing negative staining, 1 representing 1–10% of positive tumor cells and 10 representing 91–100% of positive tumor cells). For each antibody, a score that ranged from 0 to 30 was calculated as the product of staining intensity and proportion of tumor cells stained [12]. Each tumor was represented 3 times on the TMAs and the highest score was kept. For each patient, a me3/me2 score was calculated:

$$\mathrm{me}3/\mathrm{me}2\;\mathrm{score}=\log_{2}\left(\frac{\mathrm{me}3\;\mathrm{score}+1}{\mathrm{me}2\;\mathrm{score}+1}\right)$$

### GCB/ABC cell of origin (COO) subclassification

The GCB/ABC subtype was determined by cDNA-mediated Annealing, Selection, extension, and Ligation (DASL) technology based on the expression of 19 genes, as previously described [13].

### Ion torrent personal genome machine (PGM) sequencing

Genomic DNA was submitted to Next Generation Sequencing (NGS) using a laboratory-developed “Lymphopanel” set, designed to identify mutations in 34 genes important for lymphomagenesis (Supplementary Table 2). This design covers 87 703 bases and generates 872 amplicons. Amplified libraries were submitted to emulsion PCR with the Ion OneTouch™ 200 Template Kit (Life Technologies, California, USA) using the Ion OneTouch™ System (Life Technologies) according to the manufacturer's instructions. The generated Ion Sphere™ Particles (ISPs) were enriched with the Ion OneTouch™ Enrichment System and loaded and sequenced on Ion 316™ v2 Chips (Life Technologies).

### PGM data analysis

Torrent Suite™ version 4.0 (Life Technologies) software was used to perform primary analysis, including signal processing, base calling, sequence alignment to the reference genome (hg19) and generation of Binary Alignment/Map (BAM) files. BAM files were used by Torrent Suite™'s Variant Caller to detect point mutations as well as short insertions and deletions using the PGM Somatic Low Stringency profile. VCF files generated by Variant Caller were annotated by ANNOVAR [14].

Samples were considered of sufficient quality when more than 90% of targeted bases were read at least 20 times with sequencing and mapping precisions of at least Q20. Only frameshift deletions and insertions, nonframeshift deletions and substitutions, splicing, nonsynonymous, stopgain or stoploss Single Nucleotide Variations (SNVs) were kept. Variants present in dbSNP (version 138) and absent in COSMIC (version 64) were discarded, as were variants with a predictive SIFT score > 0.05 [15]. A normal probability plot defined thresholds separating true positives (confirmed by Sanger sequencing, TVC score ≥ 22) from true negatives (discredited by Sanger sequencing, TVC score < 9.5) and highlighted a gray zone (9.5 < TVC score < 22) in which variants must be confirmed by Sanger sequencing or pyrosequencing.

Further verification by Sanger sequencing was performed using a BigDye^®^ Terminator v3.1 Cycle Sequencing Kit (Life Technologies) and an ABI PRISM 3130 analyzer (Life Technologies). Primer sequences are provided in Supplementary Table 3. Further verification by pyrosequencing was performed using the PyroMark PCR kit (Qiagen, France) with internal and sequencing primers designed using PyroMark software (Qiagen). Bubble charts to visualize validated variants per patient were generated using Highcharts.com (Highsoft AS, Norway).

### Karyotyping and fluorescent *in situ* hybridization (FISH)

Cytogenetic analysis was performed according to standard techniques. Slides were RHG-banded according to Sehested [16] and karyotypes were described according to the International System for Human Cytogenetic Nomenclature. FISH using the LSI IGH/BCL2 Dual Color, Dual Fusion Translocation Probe (Vysis, Downers Grove, USA) was performed on metaphase preparations according to the manufacturer's instructions.

### Statistical analysis

All statistical analyses except kappa scores were performed using R software version 3.0.2 [17]. Kappa scores were calculated using Medcalc software version 10.0.2.0. Overall Survival (OS) was calculated from beginning of treatment to date of death or last patient follow-up. Progression-Free Survival (PFS) was calculated from beginning of treatment until disease progression, relapse, death or last patient follow-up. Log-rank tests (“survival” R package version 2.37.7) were used to assess differences in OS and PFS rates calculated by Kaplan-Meir estimates, as well as to perform univariate analysis. Multivariate analysis was performed with a Cox regression model. K-means cluster analysis was performed, with cluster number set to *k* = 2. Statistical differences between all other parameters were determined using χ^2^, Mann–Whitney, or Fisher's exact test when appropriate. *p* values < 0.05 were considered statistically significant.

## RESULTS

### Patient characteristics according to EZH2 somatic mutation status

Table 2 classifies all patients with DASL and Sanger sequencing data available based on their COO subtype and *EZH2* mutation status, and also highlights the 82 patients usable for IHC. Of the 49 GCB subtype patients, 12 were EZH2 Y641 mutant (24%), slightly higher than the original report of 22% [18]. One EZH2 mutant patient in our 100-patient cohort was of the ABC subtype, examples of which have been reported in the literature [19]. IHC-usable WT EZH2 patients were quite evenly split between ABC (*n* = 37/70) and GCB (*n* = 30/70) subtype, while all IHC-usable EZH2 mutant patients were of the GCB subtype (*n* = 12/12), as is most frequent [18, 20]. EZH2 Y641 mutations showed significant association with t(14;18) translocation in our cohort (*p* < 10^−4^), corroborating previous studies (Table 2) [20, 21].

**Table 2:** Patients according to their EZH2 mutation status

Characteristics	Total	WT EZH2	EZH2 Y641 mutant	*p*-value
Patients, *n*	92	78	14	
me3/me2 score usable, *n*	82	70	12	0.65^a^
EZH2 IHC score, *median (range)*	18 (0–30)	18 (0–30)	21 (0–27)	0.8^b^
H3K27me1 IHC score, *median (range)*	30	30	30	1^b^
H3K27me2 IHC score, *median (range)*	27 (0–27)	27 (0–27)	18 (0–27)	0.005^b^
H3K27me3 IHC score, *median (range)*	18 (0–30)	18 (0–30)	27 (0–27)	0.01^b^
me3/me2 score, *median (range)*	0 (–4.8–4.8)	–0.25 (–4.8–3.3)	0.56 (–0.56–4.8)	8.30E–05^b^
t(14;18), *n*	17	8	9	3.50E–05^a^
Age (years), *median (range)*	66 (17–87)	66 (17–87)	63 (37–77)	0.23^b^
IPI: 0–2/3–5, *n*	40/52	32/46	8/6	0.38^a^
GCB / ABC, *n*	49/43	36/39	12/1	0.005^a^

a Fisher's Exact Test

b Wilcoxon Rank Sum Test

**Abbreviations**: IPI, International Prognostic Index; GCB, Germinal Center B-Cell-like; ABC, Activated B-Cell-like

### Differential methylation levels of H3K27 are distinguishable by IHC

**Figure 1:**
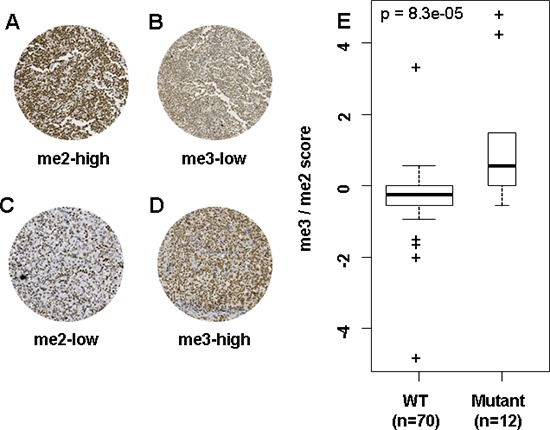
Differential IHC H3K27me2/me3 expression can distinguish WT and mutant EZH2 DLBCL **(A–D)** All images are taken at 20× magnification. **(A)** and **(B)** are images from the same WT EZH2 tumor sample. **(C)** and **(D)** are images from the same Y641 EZH2 mutant sample. IHC scores for images A–D are respectively 27/30, 9/30, 9/30 and 27/30. **(E)** is a boxplot representation of me3/me2 score according to EZH2 mutation status, showing significantly higher score in EZH2 mutant tumor samples. The width of bars in E is proportionate to sample size. *p*-values in E were calculated by a Mann–Whitney test.

FFPE samples of DLBCL placed on TMAs were used for IHC with antibodies targeting EZH2, H3K27me1, H3K27me2 and H3K27me3 (Supplementary Table 1). We used breast cancer samples of different histological subtypes, as well as DLBCL samples, as a guide to determine primary antibody concentrations and incubation times in order to observe gradients of EZH2 and H3K27 methylation IHC expression [12]. Figures 1A–1D show representative images of differential IHC expression of H3K27me2 and H3K27me3 from samples with WT or Y641 mutant EZH2. Larger versions of the same images are shown in Supplementary Figure 2. EZH2 IHC expression was also able to showcase differential levels of expression (not shown). H3K27me1 IHC expression showed high expression levels for all patients, with no differences observed (not shown).

### Patients with EZH2 Y641 mutations present distinct IHC methylation profiles

There was no significant difference in EZH2 or H3K27me1 IHC expression between patients with mutant and WT EZH2 (Table 2). Patients with EZH2 Y641 mutations presented a significantly lower H3K27me2 score (*p* = 0.005) and a significantly higher H3K27me3 score (*p* = 0.01) than patients with WT EZH2 (Table 2). Hyper-trimethylation and hypo-dimethylation in patients with EZH2 Y641 mutations is therefore evident at the IHC level. There was no significant difference in either EZH2 or H3K27me1/2/3 IHC scores between ABC and GCB subtypes (data not shown).

We thus decided to implement a score based on the ratio of me3 and me2 expression levels, in order to take into account both criteria and gain statistical strength. A logarithmic approach was used to obtain a wider distribution (me3/me2 score detailed in methods).

Y641 EZH2 mutant patients had significantly higher me3/me2 scores than patients with WT EZH2 (*p* < 10^−4^) (Figure 1E and Table 2) As me3/me2 scores for patients with WT or mutant EZH2 overlapped at zero, three distinct IHC methylation profiles emerged, centered around zero: a H3K27me3-high/H3K27me2-low profile (me3/me2 score > 0, *n* = 12/82), a H3K27me3-low/H3K27me2-high profile (me3/me2 score < 0, *n* = 38/82) and an intermediate profile (me3/me2 score = 0, *n* = 32/82). Blinded analysis by an independent pathologist without prior consultation rendered a weighted kappa score of 0.55 (Kmax = 0.8, *k* = 69% of Kmax).

The me3/me2 score is capable of distinguishing patients based on their EZH2 mutation status. Indeed, patients with EZH2 Y641 mutations mostly exhibit a H3K27me3-high/H3K27me2-low profile (*n* = 7/12), with 4/12 exhibiting an intermediate profile and 1/12 exhibiting a H3K27me3-low/H3K27me2-high profile. On the other hand, patients with WT EZH2 status are split between intermediate (*n* = 28/70) and H3K27me3-low/H3K27me2-high profiles (*n* = 37/70) (*p* = 10^−5^). The maximum accuracy of the me3/me2 score was 88%, demonstrated for a threshold > 0 (Supplementary Figure 3), leading us to merge patients with me3/me2 scores ≤ 0 into a single WT-like IHC methylation profile group, compared to the me3/me2 score > 0 mutant-like IHC methylation profile group.

### NGS mutational profiles allow more thorough understanding of IHC methylation profiles

In order to better understand the unexpected IHC methylation profiles observed for certain patients of our cohort, we performed an NGS analysis of their mutational profiles using our Lymphopanel set of genes based on literature data obtained from whole exome sequencing [22]. To this end, we sequenced all Y641 EZH2 mutant patients, as well as all WT EZH2 patients with mutant-like IHC methylation profiles. We also included 12 additional patients to extend NGS analysis to a total of 15 Y641 EZH2 mutant patients (13 GCB, 1 ABC, 1 other) and 17 WT EZH2 patients (13 ABC, 2 GCB, 2 other).

NGS results were sorted by quality scores and Sanger or pyrosequencing when possible, as described in the methods section (detailed in Supplementary Table 4). The average overall depth was 215x and the average depth for EZH2 Y646 codon was 414x. A total of 127 variants were validated in this fashion (Supplementary Table 5).

All EZH2 Y641 mutations found by Sanger sequencing were confirmed by NGS, and their VAFs as shown were calculated as the percentage of mutant reads among total number of reads. No additional EZH2 Y641 mutations were found by NGS among our cohort, and no A677 or A687 mutations were identified either. The 15 EZH2 Y641 mutants were therefore exclusively mutated at position Y641 and the 17 EZH2 WT patients were confirmed to be WT. VAFs for *EZH2* mutations calculated by pyrosequencing were highly correlated with VAFs calculated by NGS analysis (Pearson's *r* = 0.93, *p* < 10^−5^), legitimizing our NGS calculation method of VAFs for the other genes of the Lymphopanel (Supplementary Figure 4).

**Figure 2:**
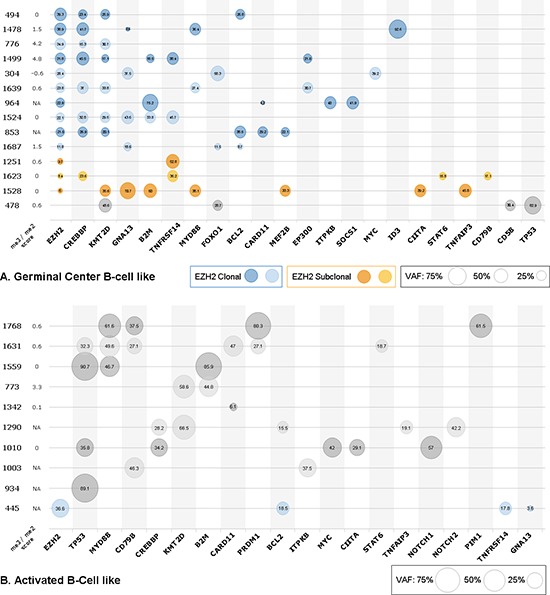
Genomic profiles of patients according to DLBCL subtype Validated variants for each patient are plotted in a bubble chart, with bubble size reflecting variant VAF, not corrected for CNVs. Patients are represented by Unique Personal Number (UPN). The value of each sample's me3/me2 score is shown, with NA corresponding to samples not present in our IHC study. Genes are ordered from most frequent to least frequent, with EZH2 first. **(A)** represents all GCB subtype patients with at least one mutation in our cohort, with clonal and subclonal EZH2 mutations outlined in blue and orange respectively. **(B)** Represents all ABC subtype patients with at least one mutation in our cohort.

Variants and their VAFs were represented in a bubble chart format according to their COO subtype (Figure 2A, 2B).

### *EZH2* mutations are majoritarily clonal in DLBCL

**Figure 3:**
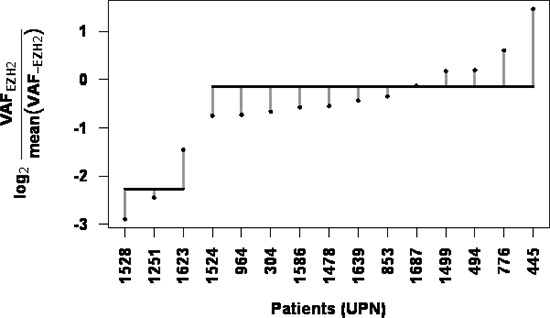
Clustering by *EZH2* mutation VAF relative to associated mutation VAFs enables subclonal mutation detection The log ratio of EZH2 VAF and average of associated mutation VAFs was calculated for each patient. *K* means clustering (*k* = 2) was performed and isolated patients 1528, 1251 and 1623 as a unique group with subclonal EZH2 mutations. Horizontal lines indicate means for each cluster and vertical dotted lines represent each point's distance to the cluster's mean.

The clonal status of *EZH2* mutations was established by comparing VAFs of EZH2 Y641 mutations with the average of those of the associated mutations in each patient. Although the direct comparison was complicated by taking into account all VAFs available (including those > 50% potentially due to CNVs), we were able to distinguish two different patterns for *EZH2* mutations. The majority of *EZH2* mutations (*n* = 12/15, 80%) represented true clonal events with similar VAFs for other genes mutated in the same sample (Figure 2A, blue bubbles and Figure 2B, patient 445). Of note, patient 1687 seems to present a clonal mutation of *EZH2* but low tumor content. True subclonal *EZH2* mutations, with lower *EZH2* mutation VAFs compared to other mutations, were found in 3/15 (20%) samples (Figure 2A, orange bubbles). *K*-means clustering was performed to separate clonal and subclonal mutations (*k* = 2) and successfully segregated these three patients (Figure 3). The percentages of clonal and subclonal EZH2 mutations in our cohort are very similar to those found in a cohort of 43 Follicular Lymphomas (FL) in a recent study by Bödör et al [23]. A recent study in DLBCL also found a similar distribution of clonal versus subclonal *EZH2* mutations [24].

Interestingly, three *EZH2* mutant GCB patients (1528, 1639 and 1478) also harbor a mutation in *MYD88*. While they remain anectodal, given the low sample size, two of these (1639 and 1478) present similar VAFs in both *EZH2* and *MYD88* mutations (38.9% and 36.4% respectively for 13944 and 23.8% and 27.4% respectively for 16995), indicating driver/clonal mutation status for both *EZH2* and *MYD88*. On the other hand, sample 1528 hosts an *EZH2* mutation with a low VAF of 6% and a *MYD88* mutation with 38.1% VAF, suggesting a driver *MYD88* mutation with a subsequent *EZH2* mutation, indicative of a secondary *EZH2* mutation acquisition in a *de novo* case of DLBCL.

### Higher number of Lymphopanel variants among the GCB subtype

On average, patients of GCB subtype (*n* = 15) presented 5.2 validated variants among the Lymphopanel genes (Figure 2A) while patients of ABC subtype (*n* = 13) presented only 2.9 validated variants (Figure 2B) (*p* = 0.02). Only 1 GCB patient (6.7%) presented no variants according to our criteria, compared to 3 ABC patients (23.1%).

Furthermore, there were 11 cases of genes displaying more than 1 variant in GCB patients (*n* = 9/15, 60%), and only 4 such cases in ABC patients (*n* = 3/13, 23.1%). Such genes in GCB patients in our cohort included *KMT2D*, *GNA13* and *CREBBP* (respectively 4, 4, and 2 cases each of patients with more than 1 variant). This mutational profile was very similar to that described in FL [23]. By contrast, such cases in ABC patients were evenly distributed among 4 genes (*PIM1*, *PRDM1*, *TNFAIP3* and *TNFRSF14*), with 1 case in each, indicating no particular variability hotspot.

### Subclonal and low-VAF *EZH2* mutations may explain unexpected WT-like IHC profiles

Potentially contributing to explain EZH2 mutant patients with WT-like IHC methylation profiles, we noted that, despite small sample size, the me3/me2 score tended to correlate with *EZH2* mutation VAF (*p* = 0.09, Pearson's *r* = 0.51). Of the five *EZH2* mutant patients presenting a me3/me2 score ≤ 0 (304, 494, 1524, 1528 and 1623), two (1528 and 1623) present low VAFs of 6% and 8.4% respectively. These two patients also exhibit a subclonal *EZH2* mutation, as determined by NGS and clustering (Figure 2A and Figure 3), suggesting that EZH2 inhibitor treatment might be less efficient.

### Associated mutations may explain unexpected IHC methylation profiles

The idea behind establishing mutational profiles for patients was to identify associated mutations, which might give reasonable cause to accept or deny treatment options, including EZH2 inhibitors, for a patient.

In our cohort, five WT EZH2 patients (1768, 1342, 1631, 773 and 478) presented a mutant-like IHC methylation profile. Four of these patients are of the ABC subtype, suggesting a potential *EZH2* mutation bypass in ABC patients. Furthermore, two of these patients (1768 and 1631) showed remarkably similar mutational profiles (Figure 2B), both of them harboring mutations in *TP53*, *MYD88*, and *PRDM1*, whereas no other ABC-subtype patient in our cohort exhibited an association of either of these mutated genes. An additional mutation in *PIM1* (patient 1768) proved interesting as well, as these were the only cases of mutations in *PRDM1* and/or *PIM1* in our cohort.

### Low EZH2 IHC expression is associated with better prognosis in ABC-DLBCL

Survival analysis was performed on the 70 patients treated with R-chemotherapy, as detailed in Table 1. The median follow-up for OS and PFS was 5.1 and 4.5 years respectively.

**Figure 4:**
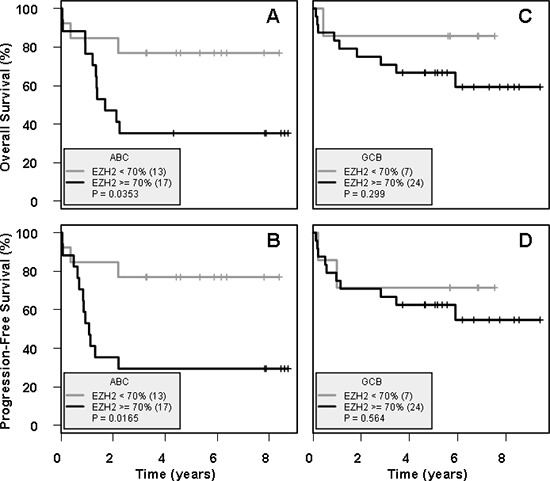
Low IHC EZH2 expression is a positive prognostic indicator in ABC-DLBCL Survival was calculated on ABC-subtype and GCB-subtype patients with R-chemotherapy treatment (*n* = 30 and *n* = 31 respectively), divided into EZH2-low (< 70%) and EZH2-high (≥ 70%) groups. **(A)** and **(B)** show OS and PFS respectively, calculated for ABC subtype patients. **(C)** and **(D)** show OS and PFS respectively, calculated for GCB subtype patients. Low EZH2 expression is associated with significantly higher OS and PFS in ABC-DLBCL patients, whereas no difference is observed in GCB-DLBCL patients.

Following the thresholds defined by a previous study [25], low EZH2 IHC expression (< 70% of tumoral cells stained) was observed in 36% of patients (55% ABC and 39% GCB), whereas high EZH2 IHC expression (≥ 70% of tumoral cells stained) was observed in 64% of patients (41% ABC and 53% GCB). In univariate analysis, low EZH2 IHC expression was significantly associated with superior OS (*p* = 0.035, OS = 77% at 3 years versus 35%) and PFS (*p* = 0.02, PFS = 77% at 3 years versus 29%) in ABC patients treated with R-chemotherapy (Figure 4A, 4B). However, in a multivariate analysis including IPI and EZH2 IHC expression in this ABC-DLBCL subgroup, neither low EZH2 IHC expression nor IPI was a statistically significant prognostic factor, with low sample number potentially responsible for this drawback. Of note, the prognostic impact of EZH2 expression was not observed in GCB patients (Figure 4C, 4D). Furthermore, no correlation was found between prognosis and IHC methylation profile in our cohort (data not shown) [23, 26].

## DISCUSSION

We have analyzed EZH2, H3K27me1, H3K27me2 and H3K27me3 IHC expression in relation to EZH2 somatic mutation status in a cohort of patients with DLBCL and shown that a simple IHC experiment is able to distinguish patients with WT EZH2 and patients with EZH2 Y641 mutations according to their me3/me2 score in the majority of cases. This result confirms the accumulation of steady state levels of H3K27me2 in WT EZH2 patients and the increase in H3K27me3 levels with lower H3K27me2 steady state levels in patients with EZH2 Y641 mutations at the IHC level. To our knowledge, this is the first such study in DLBCL. A previous study showed variable H3K27me3 and EZH2 IHC expression regardless of EZH2 mutation status in FL and H3K27me2 IHC expression was not analyzed [26].

We have also shown that no significant difference exists between patients with WT or mutant EZH2 in either EZH2 or H3K27me1 IHC expression. Lower H3K27me1 expression could have been expected in EZH2 mutant samples; however, decreased H3K27me1 in EZH2 mutant cell lines is not always observed [6] and H3K27me1 formation can also be catalyzed by noncanonical PRC2 complexes containing WT EZH1 [27]. The lack of difference in EZH2 IHC expression between patients with WT or mutant EZH2, previously shown in FL [26], confirms that the mutation mostly affects EZH2 activity, although a recent study has identified a mechanism by which it also affects EZH2 stability [28].

**Figure 5:**
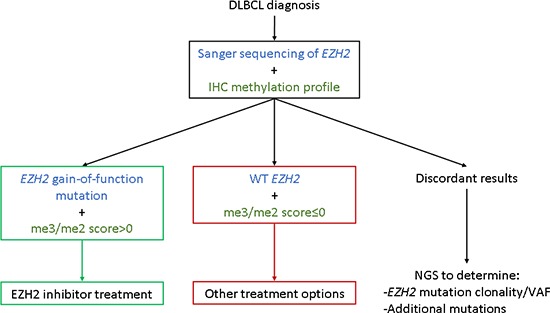
An IHC/Sanger combination approach as a decision aid for EZH2 inhibitor treatment By using an initial combination approach at time of diagnosis, three patient groups emerge, potentially simplifying EZH2 inhibitor treatment guidelines. Further analysis by NGS would thus be restricted to patients with discordant Sanger sequencing and IHC methylation profile results.

Most importantly, our me3/me2 score highlights patients with “mutant-like” and “WT-like” IHC methylation profiles. In patients with DLBCL, our IHC assay should be carried out alongside Sanger sequencing for *EZH2*. We propose that when both parameters are concordant, no further testing would be necessary: *EZH2* mutant patients with mutant-like IHC methylation profiles would be recommended for EZH2 inhibitor treatment, whereas WT *EZH2* patients with WT-like IHC methylation profiles would not. For patients with discordant IHC assay and Sanger results, NGS sequencing should be performed in order to detect *EZH2* mutation VAF or associated mutations which might justify accepting or denying EZH2 inhibitor treatment (Figure 5). Thus, a fast and readily accessible combination strategy including Sanger sequencing and an IHC assay would serve an initial filtering purpose, successfully singling out patients most likely to benefit from EZH2 inhibitor treatment, while restricting the number of patients screened by NGS for EZH2 inhibitor treatment approval.

Further comforting our hypothesis that immunohisto chemistry is a valuable tool in the determination of patients apt for EZH2 inhibitor treatment, Mccabe et al's study showed that among EZH2 mutant cell lines, H3K27me3 Western Blot levels were significantly higher in transcriptionally responsive cell lines, indicating that the association of *EZH2* mutation status and hypertrimethylation might be a more sensitive marker for EZH2 inhibitor treatment than *EZH2* mutation status only [8]. Additionally, a study showed that cell lines presenting low H3K27me2 levels in association with high H3K27me3 levels in Western Blot were more responsive to the anti-proliferative effects of EZH2 inhibitors, highlighting the importance of a mutant-like methylation profile in prospective patients [11]. IHC assays do present drawbacks in terms of inter-laboratory reproducibility, although differences could be reduced by using pixel analysis software to score staining for instance [29].

We found a minority of patients with unexpected WT-like or mutant-like IHC methylation profiles, given their mutation status, potentially predicting a respectively impaired or improved response to EZH2 inhibitor treatment. One explanation comes in the form of EZH2 mutation clonality analysis, and associated mutations might point to explanations for the remaining cases. Overall, our NGS study revealed similar mutation frequencies in genes previously analyzed in large DLBCL genomic studies [22, 30, 31] Interestingly, GCB-DLBCL with *EZH2* mutations in our cohort showed genomic profiles similar to those previously described for FL, with frequent associated mutations in *CREBBP*, *KMT2D* and *TNFRSF14*, potentially indicating a common genetic history between GCB-*EZH2* mutant-DLBCL and FL [23]. Similar data was obtained in a large DLBCL genomic study, where 5 of 7 patients with *EZH2* mutations presented associated mutations of *TNFRSF14* and 4 presented associated mutations of *KMT2D* [30].

NGS analysis has highlighted cases of interesting associated mutations in either patients with WT or Y641 mutant EZH2. Although these patients represent anecdotal evidence only at this time, they lay the groundwork for the premise that associated mutations should also be taken into account when deciding which patients to treat with EZH2 inhibitors. For instance, we detected unique mutations in *PIM1* and *PRDM1* in patients 1768 and 1631 with WT *EZH2* but mutant-like IHC methylation profiles. These genes are part of the gene network heavily affected by EZH2 binding and are involved in GC reaction [32, 33]. Interestingly, PIM1 was mutated in only one patient in our cohort, whereas previous genomic studies showed significantly higher mutation frequencies [30, 31]. While a previous study showed ABC-DLBCL cells to be refractory to EZH2 inhibitor treatment, patient-specific associated mutations such as these might modify their response and should be evaluated [34].

Furthermore, associated mutations are essential information when deciding on individual targeted therapeutic cocktails. Patients with several targetable mutations, such as patient 304 with mutations in both *EZH2* and *MYC*, might greatly benefit from an inhibitor combination approach [35, 36]. Indeed, in a recent mouse model, it was shown that only the association of an EZH2 Y641 mutation and MYC overexpression, and not the EZH2 Y641 mutation alone, led to lymphoma development [37].

Four of the five WT EZH2 patients with mutant-like IHC profiles were of the ABC subtype. While this may not be relevant for clinical trials which administer EZH2 inhibitor treatment to GCB subtype patients exclusively, it is indeed pertinent for clinical trials where the main inclusion criterion is the presence of *EZH2* gain-of-function mutations. Although rare, *EZH2* mutations in ABC subtype patients do exist, either linked to misclassification or a change in subtype during disease progression [19]. In any case, this result adds to the still-open question of the extent to which *EZH2* mutant ABC subtype patients will benefit from EZH2 inhibitor treatment.

Our me3/me2 score was not correlated with prognosis, although this was not unexpected, given previous studies showing no correlation between *EZH2* mutation status and prognosis in FL [23, 26]. On the other hand, we showed that low IHC EZH2 expression is correlated with superior OS and PFS among ABC-DLBCL patients, identifying a prognostic impact of our assay, although not present in multivariate analysis, potentially due to low sample size. A previous study in breast cancer also showed that low EZH2 expression is correlated with better Distant Disease Free Survival (DDFS) [12], corroborating our findings. On the contrary, Lee et al recently analyzed EZH2 IHC expression in a cohort of DLBCL patients of similar size and showed that high EZH2 expression was associated with superior OS, with EZH2-high ABC patients being the subgroup with the highest OS, although this finding was not quite statistically significant in multivariate analysis [25]. Compared to Lee et al, our cohort was marginally older, with a larger percentage of patients over 60 years old or with Ann Arbor stage III–IV at diagnosis. The molecular characteristics of DLBCL have indeed been shown to be age-dependent [38, 39]; however, although this might be a contributing factor, the reasons for our discrepant findings are still unclear.

EZH2 inhibitors are currently being tested in clinical trials in DLBCL as novel and promising weapons in clinicians' therapeutic arsenal. This study has shown that IHC and genomic profiles can identify patients who are most likely to benefit from treatment with EZH2 inhibitors by highlighting a specific *in vivo* H3K27me3-high/H3K27me2-low profile, determining *EZH2* mutation clonality and pinpointing associated activating mutations. Immunohistochemistry could thus serve as a convenient, fast, and easily accessible method to pre-screen patients exhibiting high me3/me2 scores for sequencing for associated mutations, thus reducing time and expenses before determining optimal, patient-specific treatment. As such, analyzing these parameters could maximize EZH2 inhibitor benefit and potentially serve to grant access to patients who would otherwise not have been considered.

## SUPPLEMENTARY FIGURES AND TABLES






