# Supplementary material for: Immunohistochemical and genomic profiles of diffuse large B-cell lymphomas: Implications for targeted EZH2 inhibitor therapy?
Source: Oncotarget. 2015 Feb 5;6(18):16712–24. doi: 10.18632/oncotarget.3154 (PMC4599301; doi:10.18632/oncotarget.3154)
Supplement: Supplementary file 1 [file oncotarget-06-16712-s001.pdf]

SUPPLEMENTARY FIGURES AND TABLES

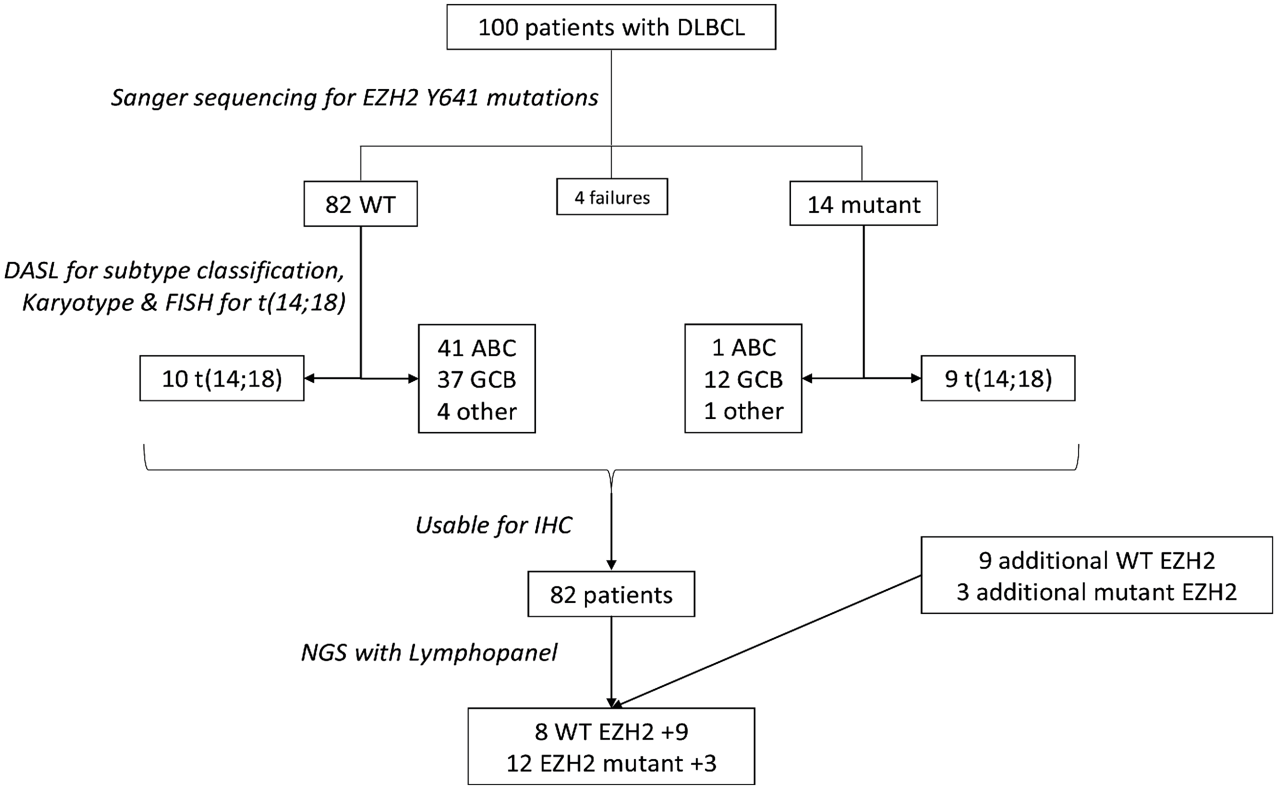

Supplementary Figure 1: Overview of cohort analysis. An initial cohort of 100 patients with DLBCL was used for Sanger sequencing of EZH2 Y641 mutations, classification by subtype using DASL and Karyotype with FISH for t(14;18) detection. Of the initial 100, 96 were successfully Sanger sequenced for *EZH2* and 82 of these were usable for IHC. NGS analysis was performed using 20 patients from our initial cohort, as well as 12 additional DLBCL patients.

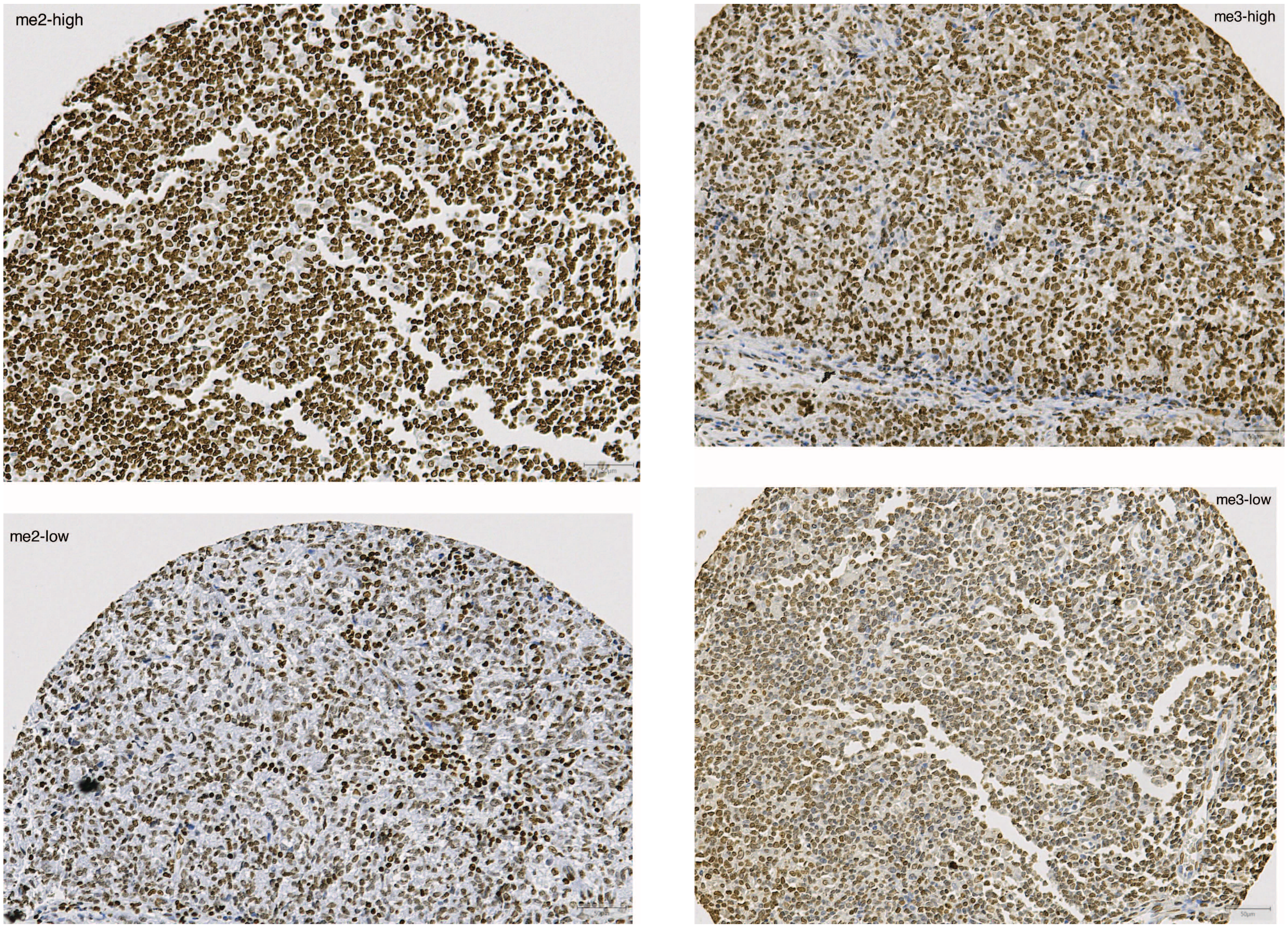

Supplementary Figure 2: Larger images of H3K27me2 and H3K27me3 IHC staining. me2-high/-low and me3-high/-low images correspond to larger version of the images shown in Figures 1 A–D.

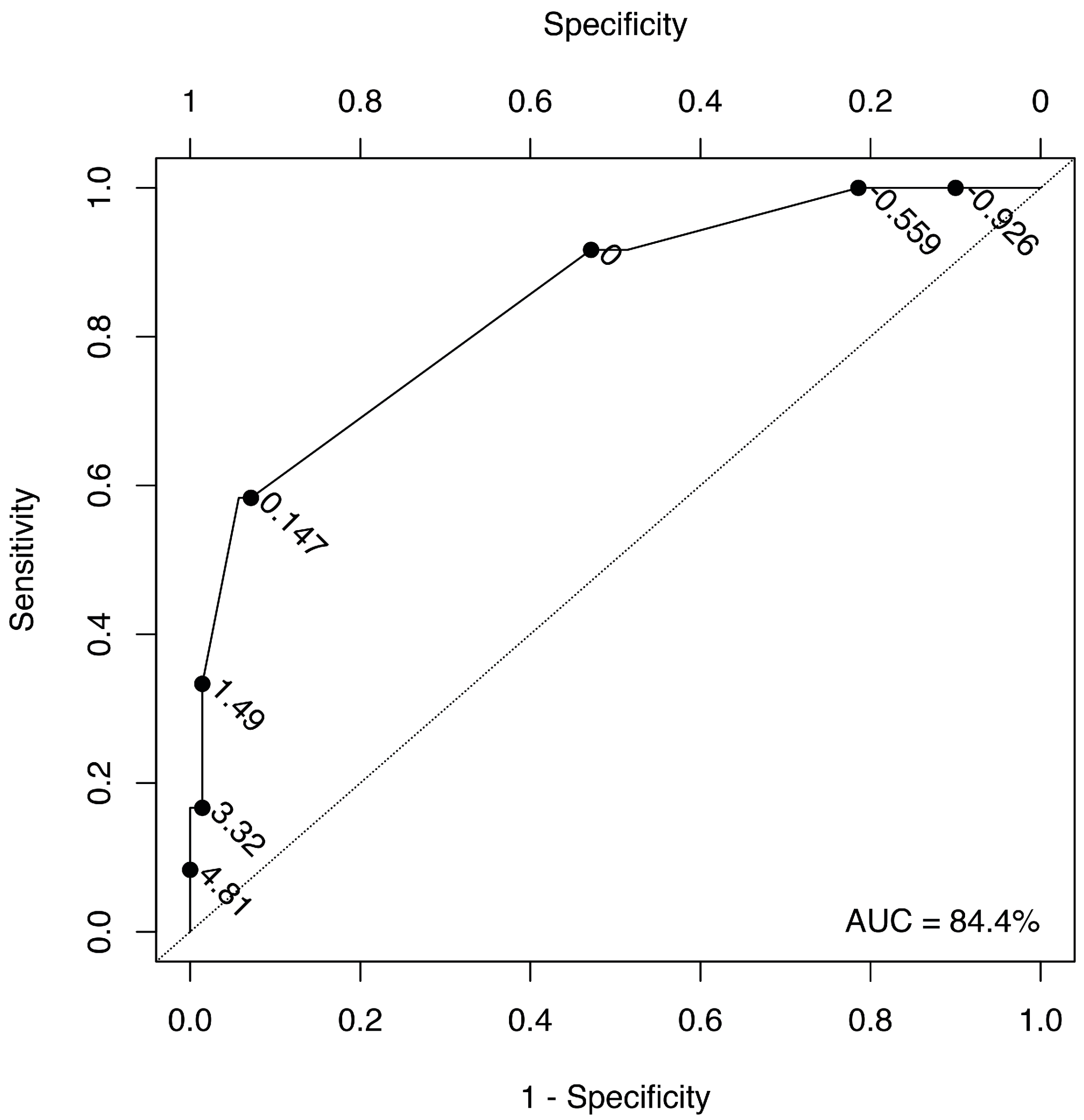

Supplementary Figure 3: Receiver Operating Characteristics (ROC) curve of me3/me2 score.

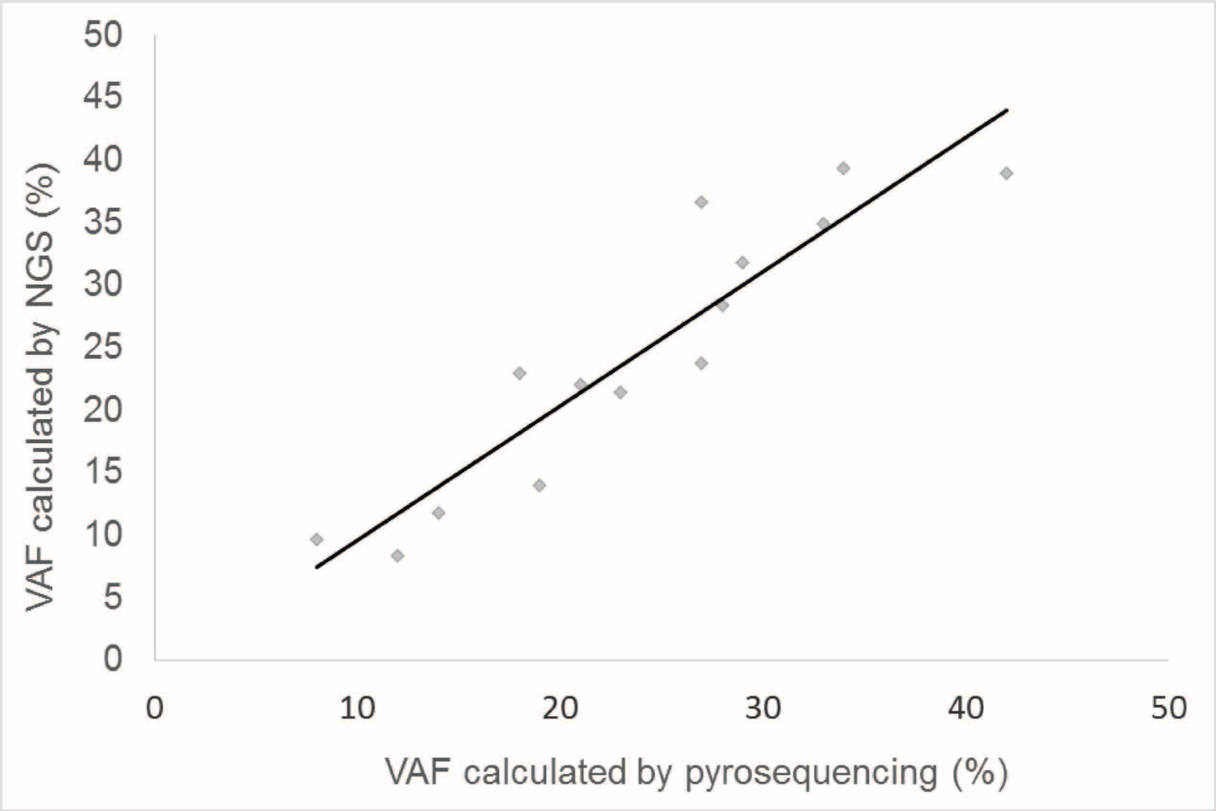

Supplementary Figure 4: *EZH2* mutation VAFs calculated by NGS and pyrosequencing are significantly correlated. 14 samples were analyzed by pyrosequencing for EZH2 Y641 mutation TAC > TTC, TAC > AAC, TAC > CAC and TAC > TGC (Y641F, Y641N, Y641H and Y641C). VAFs calculated by both methods were highly correlated (Pearson's $r = 0.93$, $p < 10^{-5}$).

Supplementary Table 1: Primary antibody characteristics

Antigen	Antibody/Clone	Dilution	Incubation Time	Source	Reference
**EZH2**	Rabbit monoclonal D2C9	1:200	1 hour	Cell Signaling Technology, Massachusetts (USA)	5246
**H3K27me1**	Rabbit polyclonal	1:150	Overnight	Abcam, Cambridge (UK)	ab113671
**H3K27me2**	Rabbit polyclonal	1:3000	1 hour	Abcam	ab24684
**H3K27me3**	Mouse monoclonal	1:500	1 hour	Abcam	ab6002

Supplementary Table 2: Overview of the lymphopanel set used for NGS analysis

Gene	Transcript Reference	Hotspot/Exons partially sequenced	Chromosomal location	Size sequenced (bp)
**B2M**	NM_004048	Hotspots exons 1 & 2, exon 3	15q21.1	360
BCL2	NM_000633	Hotspot exon 2	18q21.33	585
BRAF	NM_004333	Exon 15	7q34	119
CARD11	NM_032415	Coiled-coil domain exons 4–9	7p22.2	1121
CD58	NM_001779	Exons 1–6	1p13.1	753
CD79A	NM_001783	ITAM domain exons 4 & 5	19q13.2	183
CD79B	NM_000626	ITAM domain exons 5 & 6	17q23.3	141
CDKN2A	NM_000077 + NM_058195 + NM_058197 + NM_001195132	Exons 1, 2A, 2B, 3, 4 & 5	9p21.3	1,737
CDKN2B	NM_004936 + NM_0078487	Exons 1A, 1B & 2	9p21.3	1289
CIITA	NM_000246	Exons 1–19	16p13.13	3393
CREBBP	NM_004380	Exons 1–31	16p13.3	7323
EP300	NM_001429	Exons 1–31	22q13.2	7245
EZH2	NM_004456	SET domain, hotspots exon 16 & 18	7q36.1	177
FOXO1	NM_002015	Hotspots exon 1 & FH domain exon 2	13q14.11	780
GNA13	NM_006572	Exons 1–4	17q24.1	1134
ID3	NM_002167	Exons 1 & 2	1p36.12	360
IRF4/MUM1	NM_002460	Exons 2–9	6p25.3	1356
ITPKB	NM_002221	Exons 2–8	1q42.12	2830
**KMT2D/MLL2**	NM_003482	Exons 1–54	12q13.12	16614
MEF2B	NM_001145785	Exons 2–9	19p13.11	1107
MFHAS1	NM_004225	Exons 1–3	8p23.1	3159
MYC	NM_002467	Exons 1–3	8q24.21	1365
MYD88	NM_001172567	Exons 2–5	3p22.2	587
NOTCH1	NM_017617	PEST domain exon 34	9q34.3	1488
NOTCH2	NM_024408	Exons 26–28 & 34 (HD/PEST domains)	1p12–p11.2	2091
PIM1	NM_002648	Exons 1–6	6p21.2	942
PRDM1/BLIMP1	NM_001198	Exons 1–7	6q21	2478
SOCS1	NM_003745	Exon 2	16p13.13	636

(*Continued*)

Gene	Transcript Reference	Hotspot/Exons partially sequenced	Chromosomal location	Size sequenced (bp)
STAT6	NM_001178078	Exons 9–14 (DNA binding domain hotspot)	12q13.3	795
TCF3	NM_001136139	B-HLH domain of E47 isoform exons 17 & 18	19p13.3	370
TNFAIP3	NM_006290	Exons 2–9	6q23.3	2373
TNFRSF14	NM_003820	Exons 1–8	1p36.32	852
TP53	NM_000546	Mutation hotspots exons 4–10	17p13.1	1004
XPO1	NM_003400	Exons 15–18	2p15	640

Supplementary Table 3: Overview of sample and variant quality controls for validation

Total samples sequenced	32
Samples eliminated due to low quality	1
Total variants of accepted types, dbSNP & Cosmic status and SIFT scores	301
Variants in gray zone of quality score	50
Percentage of validated variants in gray zone	8.6
Total variants validated by quality score +/− Sanger or pyrosequencing	127

Supplementary Table 4: Primer sequences used for Sanger sequencing

Supplementary Table 5: Overview of the validated variants
